# Genetic trends in the Kenya Highland Maize Breeding Program between 1999 and 2020

**DOI:** 10.3389/fpls.2024.1416538

**Published:** 2024-07-01

**Authors:** Dickson O. Ligeyo, Edward Saina, Bornface J. Awalla, Clay Sneller, Walter Chivasa, Lennin Musundire, Dan Makumbi, Mable Mulanya, Dragan Milic, Samuel Mutiga, Abraham Lagat, Biswanath Das, Boddupali M. Prasanna

**Affiliations:** ^1^ Department of Food Crops and Research Institute, Kenya Agricultural and Livestock Research Organization, Kitale, Kenya; ^2^ Department of Horticulture and Crop Science, The Ohio State University, Wooster, OH, United States; ^3^ Global Maize Program, International Maize and Wheat Improvement Center, (CIMMYT), Nairobi, Kenya; ^4^ Integrated Breeding Platform (IBP), Nairobi, Kenya

**Keywords:** maize, genetic gain, Kenya national breeding program, highland ecology, breeding

## Abstract

Optimization of a breeding program requires assessing and quantifying empirical genetic trends made through past efforts relative to the current breeding strategies, germplasm, technologies, and policy. To establish the genetic trends in the Kenyan Highland Maize Breeding Program (KHMP), a two-decade (1999–2020) historical dataset from the Preliminary Variety Trials (PVT) and Advanced Variety Trials (AVT) was analyzed. A mixed model analysis was used to compute the genetic gains for traits based on the best linear unbiased estimates in the PVT and AVT evaluation stages. A positive significant genetic gain estimate for grain yield of 88 kg ha^−1^ year^−1^ (1.94% year^−1^) and 26 kg ha^−1^ year^−1^ (0.42% year^−1^) was recorded for PVT and AVT, respectively. Root lodging, an important agronomic trait in the Kenya highlands, had a desired genetic gain of −2.65% year^−1^ for AVT. Results showed improvement in resistance to Turcicum Leaf Blight (TLB) with −1.19% and −0.27% year^−1^ for the PVT and AVT, respectively. Similarly, a significant genetic trend of −0.81% was noted for resistance to Gray Leaf Spot (GLS) in AVT. These findings highlight the good progress made by KHMP in developing adapted maize hybrids for Kenya’s highland agroecology. Nevertheless, the study identified significant opportunities for the KHMP to make even greater genetic gains for key traits with introgression of favorable alleles for various traits, implementing a continuous improvement plan including marker-assisted forward breeding, sparse testing, and genomic selection, and doubled haploid technology for line development.

## 1 Introduction

Maize (*Zea mays* L.) is the primary cereal crop in Kenya, covering approximately 2.1 million ha of land, whereas over 75% of the area is devoted to cereal production (FAOSTAT, 2013). The majority (~81%) of the maize farmers in Kenya are small-scale growers who cultivate the crop on less than 2 ha of land. With a per capita consumption range of 98 kg–103 kg, maize is a major source of staple food calories, accounting for 65% and 36% of the total caloric intake by the Kenyan population (De Groote et al., 2005; Kirimi et al., 2011; Marenya et al., 2021). Maize productivity increased from 1.3 t ha^−1^ in 1979 to 1.5 t ha^−1^ in 2021, but the current yield is still far below the global average of 5.8 t ha^−1^ (FAOSTAT, 2013). Furthermore, the current annual grain production of 3.3 million tons only caters for approximately 75%–80% of the country’s demand (Erenstein et al., 2022). The low maize production in sub-Saharan Africa (and Kenya in particular) stems from the inability of the small-scale farmers to adopt good agronomic practices that would optimize the performance of the improved varieties across agroecologies and overcome the Genotype × Environment × Management (G × E × M) interactions (Dao et al., 2015; Cairns et al., 2021). Genetic innovations for enhanced crop resilience could reduce the G × E × M effects, increasing maize production by up to 25% (Cairns and Prasanna, 2018).

Deploying climate-resilient crops and climate-smart agricultural practices is critical for closing yield gaps and reducing the high risk and vulnerability, particularly for smallholder farmers (Cairns et al., 2021). To minimize G × E × M effects, the Kenyan breeding programs and their partners have focused on development of adapted and resilient maize varieties for cultivation across different agroecological zones, including the moist transitional highlands, highland tropics, mid-altitudes, moist transitional, dry-transitional, dry mid-altitudes, and tropical lowlands agroecologies (Jatzold and Kutsch, 1982; Miruka et al., 2012; De Groote et al., 2023). The highland agroecological zone (1,700 m to 2,300 m above sea level, masl; mean annual rainfall of 1,000 mm to 1,800 mm) is important to Kenyan food security because it is less prone to drought, heat, and flooding than other zones (Kostandini et al., 2013). The highlands agroecological zone covers 12% of the maize area and produces approximately one-third of the maize grain in Kenya (De Groote and Omondi, 2023). A recent analysis of the potential effects of climate change shows that maize yields will reduce by 10%–20% in Sub-Saharan Africa (SSA) by 2050 but the yield potential in the highlands of Kenya and Ethiopia will increase due to an increase in temperature (Philip et al., 2010; Cairns et al., 2013; Tesfaye et al., 2015). Unfortunately, the area classified as highlands in Kenya will decrease by 35% due to climate change (Tesfaye et al., 2015). Increasing maize yields in farmers’ fields requires an integrated approach of various interventions, including increased adoption of new climate-resilient varieties, access by smallholders to the seed of improved varieties, increased fertilizer use, adoption of new agronomic management practices, and supportive policies (Cairns et al., 2021).

The Kenyan Highland Maize Program (KHMP) focuses on developing improved varieties for the highlands and is one of the six maize improvement initiatives of the Kenya Agricultural and Livestock Research Organization (KALRO) (Miruka et al., 2012; Schroeder et al., 2013). The program began with informal maize breeding operations in the 1930s at Njoro in Nakuru and was streamlined when the operations were relocated to Kitale in 1955 (Gerhart 1975). The initial core activities involved collections of suitable local white kernel parental germplasm, which was used to develop the first two synthetic varieties, Kitale Synthetic I (KSI) and Kitale Synthetic II (KSII), at Kitale in 1955 (Harrison, 1970). To strengthen the breeding pool, additional new genetic stocks were also introduced from similar agroecologies of Central and Southern America (e.g., the Ecuadorian landrace, Ecuador 573 and Costa Rica 296) through the International Maize and Improvement Center (CIMMYT) (Harrison, 1970). The imported germplasm was used to develop the first maize hybrids (e.g., H611 developed from Ecuador 573 × KSII and released in 1964), which had higher heterosis for grain yield (140%) than the earlier synthetic varieties (Darrah et al., 1972, 1978). KHMP uses Ecuador 573 and KSII as the heterotic groups in well-defined hybrid development pipelines (Wende et al., 2018).

In the last three decades, 394 improved maize varieties have been released in Kenya, with 87 (22%) coming from the KALRO maize breeding programs; 17 of the 394 varieties (4%) originate from the KHMP (KEPHIS, 2023). A high (80%–95%) rate of adoption of certified seed in the Kenyan highlands has been achieved through varietal development, advocacy, and marketing programs, which are implemented by KALRO, the Kenya Seed Company Limited (a parastatal with a mandate for certified seed production and marketing), international partners, and other private seed companies (Smale and Olwande, 2011; Wang et al., 2017).

Assessing genetic trends for the major traits can provide insights into optimizing the pipelines to enhance genetic gains for traits of interest to a breeding program (Khanna et al., 2022). Breeders can assess the response or gain in a breeding program by using “the breeder’s equation” (Eberhart, 1970; Xu et al., 2017). The breeder’s equation estimates potential genetic gains in a population undergoing cycles of selection and recombination and is based on estimates of variances (Falconer and Mackay, 1996; Rutkoski et al., 2022). Recent interest has been in assessing the genetic gains in breeding programs that are not entirely focused on population improvement through selection cycles (Badu-Apraku et al., 2015). There are limited studies on genetic gains in the highlands breeding programs in Africa. A previous genetic gain report for the KHMP was published in the 1970s based on older highland maize genotypes, some of which were progenitor populations of the germplasm used in this study (Eberhart and Harrison, 1973). Kebede et al. (2020) reported genetic gains of 16.26 kg ha^−1^ year^−1^ for GY in Ethiopia’s highland maize breeding program. However, no recent studies have been done on KHMP. Therefore, this study aims to assess the historical genetic gains based on the trends of the changes in the genetic values for GY, yield-related traits, and major diseases over a 22-year breeding period at the KHMP and to utilize the results to identify areas of improvement, including potential optimization of the breeding pipeline.

## 2 Materials and methods

### 2.1 Description of the germplasm and trials

Data on germplasm developed by the KHMP, which included three-way hybrids (70%), top cross hybrids (15%), single-cross hybrids (15%), and commercial check hybrids, evaluated in trials between 1999 and 2020, was used in the study. Approximately 10% of the evaluated genotypes had parental components derived from the doubled haploid (DH) technology. In contrast, the rest of the genotypes were developed using the pedigree breeding method in which selected parental inbred lines were hybridized to generate F_1_ hybrid seed and individual plants were selected from F_2_ and advanced up to F_6_ prior to crossing with two single cross testers representing the Kitale synthetic and Ecuador composite heterotic groups, respectively, to generate three-way hybrids. Single-cross hybrids were generated from hybrid combinations between inbred lines from the pedigree breeding method and DH technology. These single-cross hybrids were also used as parental combinations to generate double-cross hybrids. Top crosses were generated from cross combinations between open-pollinated varieties, inbred lines developed within the KHMP, and introductions from partners, namely, Ethiopia’s Highland maize breeding program and Uganda’s National Agricultural Research Organization (NARO)’s Highland maize breeding program and CIMMYT’s Global Maize Program. Various local and commercial check varieties were used in the trials for years, but common checks were maintained to provide connectivity and allow genetic gain estimates. The number of entries evaluated across the years varied, with most of the genotypes in the Preliminary Variety Trials (PVT, 86%) and the Advanced Variety Trials (AVT, 80%) being tested in just 1 year, 12% and 15% in the second year for the PVT and AVT, respectively; 2% and 5% evaluated in the third year for the PVT and AVT, respectively; only 1% of the entries tested in the fourth year for the AVT (**Table 1**).

**Table 1:** Summary of the composition of the Kenya highland maize Preliminary Varietal Trials (PVT) and Advanced Varietal Trials (AVT).

Factors	PVT	AVT
Number of years	13	20
Number of experiments	55	146
Number of replications	3	4
Number of entries per year	36	25
Total number of entries	394	399
Total number of breeding lines	392	396
Number of breeding lines tested for 1 year	339	319
Trial design layout	Alpha lattice	Alpha lattice
Number of breeding lines tested for 2 years	47	58
Number of breeding lines tested for 3 years	6	14
Number of breeding lines tested for 4 years	0	5

### 2.2 Experiment design and trial management

**Figure 1:**
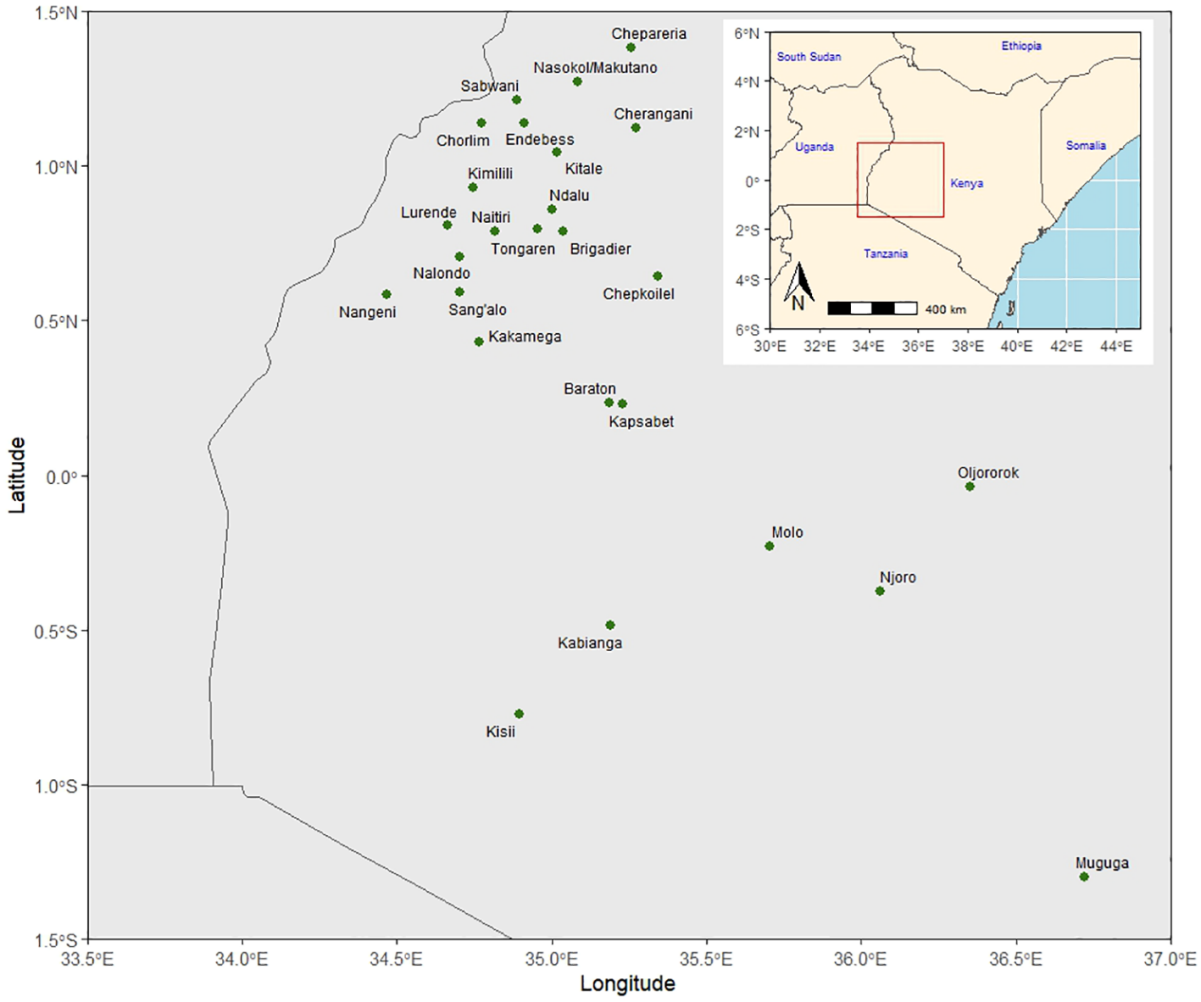
Kenya highland multi-location trial sites for preliminary and advanced maize varietal trials.

The KHMP uses the product development pipeline, which involves the stage gate advancement protocol across the different stages (from PVT to AVT). Each advancement stage is defined relative to activities and key decisions based on defined threshold values for key traits (**Supplementary Table 1**) taken between the stages. An alpha lattice design was used in the PVT and AVT experiments. In the PVT, 36 entries year^−1^ and 394 entries with three replications over 13 years were evaluated in 55 experiments. Similarly, in AVT, 25 entries year^−1^ and 399 entries, with four replications over 22 years, were assessed in 146 experiments between 1999 and 2020 across 27 locations in the Highlands of Kenya (**Table 2**; **Figure 1**). The experiments were planted in three-row plots, 3-m-long rows with 0.75-m row spacing and 0.30-m in-row spacing, with a final plant density of approximately 44,444 plants ha^−1^. AVT were planted in two-row plots, 5-m-long rows with row spacing of 0.75-m and 0.25-m in-row spacing, with a final plant density of approximately 53,333 plants ha^−1^. The trials were fertilized using basal fertilizer (18:46:0–N:P: K) at an average rate of 190 kg ha^−1^ at planting and top dressing using Top CAN (26% N) at 190 kg ha^−1^ split into two equal applications of 95 kg ha^−1^, with the first half applied at the early vegetative stage and the second at the booting (pre-flowering) stage as recommended in the PVT and AVT. The major traits of focus were GY (kg ha^−1^), plant and ear height (PH and EH) (cm), root and stalk lodging (RL and SL) (%), ear rot (ER) (%), husk cover (HC) (%), and disease severity scores (1–5) for Grey leaf spot (GLS) and Turcicum leaf blight (TLB). Hand weeding was used to keep the trial area weed-free during the crop-growing period.

**Table 2:** Agro-ecology details of Kenya highland multi-location trial sites (1999–2020).

Location	Latitude	Longitude	Altitude (masl)	Annual average temperature (°C)		Mean rainfall (mm)	Average annual RH
				Min	Max		
Baraton	0.236	35.185	1,975	12	23	1,648	62
Brigadier	0.787	35.038	1,830	10	30	1,100	78
Chepareria	1.381	35.256	1,723	14	30	1,134	71
Chepkoilel	0.643	35.343	2,205	10	26	1,103	75
Cherangani	1.123	35.273	1,870	11	29	1,300	71
Chorlim	1.137	34.773	2,400	10	29	1,450	71
Endebess	1.139	34.910	1,900	11	29	1,280	69
Kabianga	−0.486	35.188	1,780	10	30	1,300	81
Kakamega	0.430	34.765	1,540	17	35	1,395	69
Kapsabet	0.229	35.230	1,950	9	29	1,600	78
Kimilili	0.929	34.746	1,723	11	29	1,500	75
Kisii	−0.773	34.894	1,879	15	27	1,500	62
Kitale	1.043	35.017	1,900	11	29	1,450	71
Lurende	0.806	34.663	1,700	11	29	1,500	77
Makutano-Pokot	1.250	35.833	2,025	14	31	1,429	75
Molo	−0.228	35.705	2,482	12	26	1,430	70
Muguga	−1.298	36.721	2,050	11	30	1,200	74
Naitiri	0.787	34.817	1,740	11	29	1,100	78
Nalondo	0.707	34.704	1,370	11	29	1,300	73
Nangeni	0.585	34.468	1,340	16	33	1,300	73
Nasokol/Makutano	1.271	35.082	2,050	11	29	1,429	75
Ndalu	0.859	35.001	1,820	11	29	1,450	71
Njoro	−0.374	36.060	1,800	7	31	1,000	61
Oljororok	−0.039	36.351	2,400	7	31	1,500	73
Sabwani	1.214	34.889	1,880	10	30	1,034	65
Sang’alo	0.591	34.701	1,420	16	33	1,500	73
Tongaren	0.798	34.954	1,713	11	29	1,100	73

Masl, meters above sea level; °C, degrees Celsius; mm, millimeters; RH, relative humidity (%).

#### 2.2.1 Trait measurements

Data were collected at all the sites applying standard procedures used at the International Maize and Wheat Improvement Center (CIMMYT 1985) for the following traits: (a) plant and ear height was measured 2–3 weeks after flowering on 5–10 selected plants, measuring the distance from the plant base to the point where the tassel starts to branch and the distance in centimeters from the plant base to the node bearing the uppermost ear, respectively; (b) percentage stalk and root lodging was recorded as a percentage of plants per plot that had their stems broken below the ears but not above the ears and percentage of plants per plot which had their stems inclined at least 30° or more from the perpendicular at the base of the plant where the root zone stars, respectively; (c) ear rot (ER): incidence of ear and kernel rots caused by *Diplodia* spp., *Fusarium* spp., or *Gibberella* spp., counted and expressed as a percentage of the number of harvested ears; (d) husk cover (HC): percentage number of ears in each plot that had any portion of the ear exposed relative to the total number of ears per plot; (e) severity of GLS and TLB diseases were scored 3 weeks after flowering on a score scale of 1–5 (where 1 = no visible leaf damage; 5 = severe damage of >50% of the leaf area) (Ngwira and Pixley, 2000; Bankole et al., 2022). All plants were hand-harvested and shelled, and grain weight was measured in grams (g). Shelled grain weight (g) was used to estimate GY adjusted to 12.5% moisture content at harvest and expressed to kg ha^−1^.

### 2.3 Data analysis

The analysis of variance (ANOVA) for individual environments was carried out for GY, agronomic traits, and disease severity using a model in which genotypes and replications were fitted as random effects. An analysis of variance was performed within each experiment using the model (Equation 1):


(1) $$y_{ij}=\mu +g_{i}+r_{j}+e_{ij}$$

where *y_ij_* is the mean phenotypic value of the trait within an experiment, *g_i_* is the random effect of the *i* ^th^ genotype with $g_{i}∼N(0,\;\sigma_{g}^{2})$ , *r_j_* is the random effect of the *j* ^th^ replication with $r_{j}∼N\left(0,\;\sigma_{r}^{2}\right)$ , and $e_{ij}$ is the residual error with $e_{ij}∼N\left(0,\;\sigma_{e}^{2}\right)$ .

Analysis of variance was conducted over all experiments within each year using the model (Equation 2):


(2) $$y_{ijk}=\mu +g_{i}+l_{k}+r{\left(l\right)}_{jk}+gl_{ik}+\;e_{ijk}$$

where $y_{ijk}$ is the mean phenotypic value of the trait within an experiment within a year, $l_{k}$ is the random effect of the *k* ^th^ location with $l_{k}∼N(0,\;\sigma_{l}^{2}$ ), gl_ik_ is the interaction of the i^th^ genotype with the k^th^ location with $gl_{ik}∼N(0,\;\sigma_{gl}^{2}$ ), *r*(*l*)*_jk_*. is the random effect of the *j* ^th^ replication nested in the k^th^ location with $r{\left(l\right)}_{jk}∼N\left(0,\;\sigma_{r\left(l\right)}^{2}\right)$ , and $e_{ijk}$ is the residual error with $e_{ijk}∼N\left(0,\;\sigma_{e}^{2}\right)$ .

GY (kg ha^−1^) and agronomic performance of genotypes evaluated in the current study highlighted variation among the genotypes (**Table 1**) in the PVT and AVT experiments. Broad sense heritability was determined for all traits within each experiment. The variance components from the above models were used to calculate entry mean heritability within each experiment as (Equation 3):


(3) $$H=\frac{\sigma_{g}^{2}}{\sigma_{g}^{2}+\langle \frac{\sigma_{e}^{2}}{r}\rangle }$$

where $\sigma_{e}^{2}$ is the genetic variance, $\sigma_{e}^{2}$ is the error variance, and *r* is the number of replications.

Similarly, the mean broad-sense heritability within a year was calculated as (Equation 4):


(4) $$H=\frac{\sigma_{g}^{2}}{\sigma_{g}^{2}+\langle \frac{\sigma_{gl}^{2}}{l}\rangle +\langle \frac{\sigma_{e}^{2}}{lr}\rangle }$$

where $\sigma_{gl}^{2}\;$ is the genotype × location variance, l is the number of locations for the trial, and *r* is the number of replications.

### 2.4 Genetic gain estimates

The rate of genetic gain using data from the KHMP PVT (2003–2020) and AVT (1997–2020) trials that were conducted across Kenya highland environments (**Figure 1**) were estimated. To compute genotypic Best Linear Unbiased Estimates (BLUEs) for use in subsequent regression models, a mixed model, which included entries (genotypes) as fixed effects was fitted using the model (Equation 5):


(5) $$y_{ijklm}=u+g_{i}+y_{j}+l_{k}+r{\left(l\right)}_{lk}+gy_{ij}+gl_{ik}+gly_{ijk}+c_{m}+\;e_{ijkl}$$

where y_ijklm_ is the phenotype of the i^th^ entry in the j^th^ year in the k^th^ location and the l^th^ replication, g_i_ is the fixed effect of the i^th^ genotype, y_j_ is the effect of the j^th^ year with $y_{j}∼N(0,\;\sigma_{y}^{2}$ ), l_k_ is the effect of k^th^ location with $l_{k}∼N(0,\;\sigma_{l}^{2}$ ), r(l)_lk_ is the effect of the l^th^ rep nested in the k^th^ location with $r{\left(l\right)}_{lk}∼N(0,\;\sigma_{r\left(l\right)}^{2}$ ), gy_ij_ is the interaction of the i^th^ genotype with the j^th^ year with $gy_{ij}∼N(0,\;\sigma_{gy}^{2}$ ), gl_ik_ is the interaction of the i^th^ genotype with the k^th^ location with ), gly_ijk_ is the interaction of the i^th^ genotype with the j^th^ year and k^th^ location with $gly_{ijk}∼N(0,\;\sigma_{gly}^{2}$ ), c_m_ is the fixed effect of control (1 for a check, 0 for a breeding line), and e_ijklm_ is the error with $e_{ijklm}∼N(0,\;\sigma_{e}^{2}$ ). Genotype and check effects were considered fixed, and all other effects were considered random.

A concept referred to as retrogressive analysis (Ci et al., 2011; Badu-Apraku et al., 2015) was used to estimate the genetic gain trends (hereinafter referred to as genetic gains) for the key traits in trials evaluated between 1999 and 2020 within the KHMP. In the model, the best linear unbiased estimates (BLUEs), which were considered to be the genetic gain values of individual maize traits, were generated in a mixed statistical analysis model (Henderson, 1975). The genetic gain was estimated by regressing each genotype’s estimated value in the first year the genotype was tested (FYT) in the 2003 to 2020 data set for PVT and in the 1997 to 2020 data set for AVT. The FYT was defined as the base year the genotype entered the trial (PVT or AVT). The genetic gain was reported based on the direction of the trend (slope) and was declared to be significant if the probability of the slope was less than 0.05 and had an R^2^ greater than 0.05.

## 3 Results

### 3.1 Broad-sense heritability and quality of data in the experiments

The broad-sense heritability (H) estimates expressed as a percentage were classified according to Robinson et al. (1949) into three classes: low 0%–30%, medium 31%–60%, and >60% as high. The results revealed that in the PVT, GY and HC had medium H (**Table 3**). In this trial series, 25% and 11% of the experiments had H <0.2 for GY and HC, respectively. A higher proportion of experiments with H >0.2 was observed for the other agronomic traits and foliar diseases. In the AVT GY, EH, PH, SL, and HC had medium H (**Table 4**). Low H^2^ was recorded for RL, foliar disease severity scores for GLS TLB and ER in both AVT and PVT (**Tables 3**, **4**). Experiments with H > 0.2 in the PVT (75%) and AVT (84%) for GY were used to compute the best linear unbiased estimates (BLUEs) for each genotype referred to as genetic values in this study. Similarly, for agronomic traits, data sets with H > 0.2 ranged from 34% to 89% in the PVT and 35%–90% for agronomic traits and disease severity were used to compute BLUEs.

**Table 3:** Summary of maize trait values and heritability within the experiment and years for the PVT conducted between 1999–2020.

Trait	Years (*n*)	Experiments (*n*)	Trait values		Heritability (*H*)	
			Mean	Range	Mean	% <0.2
Grain yield (kg ha^−1^)	12	52	4,530	1,700–10,100	0.40	25.0
Ear height (cm)	13	50	191	127–249	0.23	53.5
Root lodging (%)	12	47	0.5	0–11	0.13	66.0
Stalk lodging (%)	12	47	10.2	1–35	0.26	41.3
Ear rot (%)	13	53	7.8	0–15	0.29	41.5
Poor husk cover (%)	13	53	4.9	1–14	0.45	11.3
Grey leaf spot (1–5)	11	24	2.3	1–3	0.16	54.2
Turcicum leaf blight (1–5)	13	28	2.2	1–4	0.20	60.7

An experiment was defined as a year/location combination.

**Table 4:** Summary of maize trait values and heritability within the experiments and years for the AVT conducted between 1999 and 2020.

Trait	Years (*n*)	Experiments (*n*)	Trait values		Heritability (*H*)	
			Mean	Range	Mean	% <0.2
Grain yield (kg ha^−1^)	18	127	6,170	900–14,100	0.51	15.7
Plant height (cm)	20	137	290	156–401	0.45	17.5
Ear height (cm)	20	138	175	26–421	0.52	10.1
Root lodging (%)	20	139	1.8	0–21	0.16	64.7
Stalk lodging (%)	19	131	7.6	0–86	0.37	26.0
Ear rot (%)	20	137	3.3	0–39	0.26	39.4
Poor husk cover (%)	19	139	4.3	0–23	0.49	15.1
Grey leaf spot (1–5)	19	75	1.9	1–3	0.27	56.0
Turcicum leaf blight (1–5)	20	79	1.7	1–3	0.28	45.6

An experiment was defined as a year/location combination.

### 3.2 Genetic trends for grain yield, agronomic traits, and diseases

**Figure 2:**
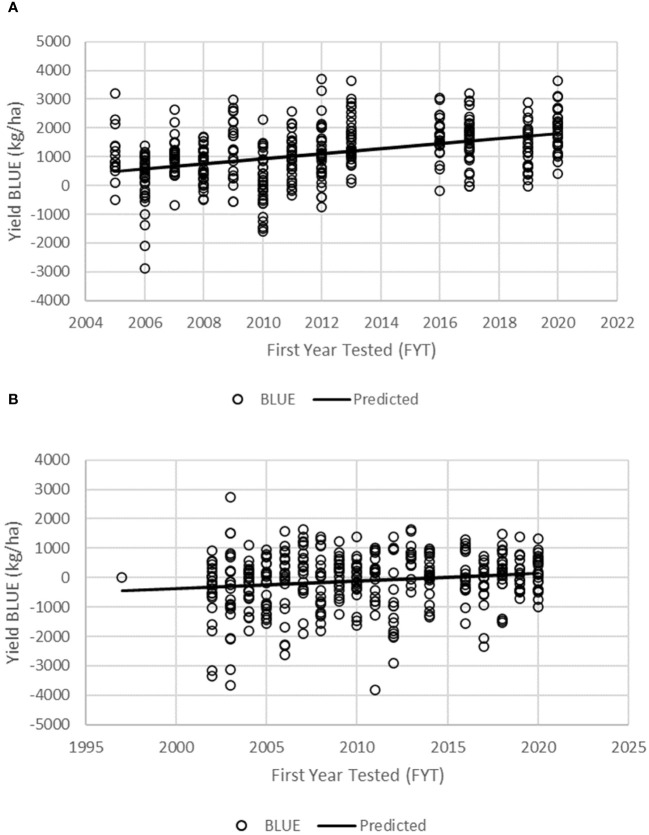
Regression of grain yield BLUEs of maize inbred lines onto the first year of testing for all genotypes in the KMHP preliminary varietal trial (**A**, PVT) and advanced varietal trial (**B**, AVT).

The BLUEs were used in linear regression analysis to assess the genetic gain values for GY and other agronomic traits in the PVT and AVT. The regression model accounted for 16.5% and 2.4% of the variation for GY for genotypes in the PVT and AVT, respectively (**Table 5**; **Figure 2**). There was significant (p < 0.05) positive annual genetic gain in GY for the genotypes in the PVT and AVT. The PVT genotypes had a mean GY of 4,530 kg ha^−1^ and an annual genetic gain estimate of 88 kg ha^−1^ or 1.94% year^−1^ (**Table 5**; **Figure 2**). In the AVT, genotypes had a higher mean GY than the PVT genotypes (6,170 kg ha^−1^) but a smaller annual genetic gain estimate of 26 kg ha^−1^ or 0.42% year^−1^.

**Table 5:** Genetic trends in maize trait values for grain yield, agronomic traits, and disease resistance in PVT and AVT between 1999 and 2020.

Trait	PVT				AVT			
	Magnitude* of gain in trait value	Annual gain in trait value (%)^α^	*P-value*	*R^2^*	Magnitude of gain in trait value	Annual gain in trait value (%)	*P-value*	*R^2^*
Grain yield (kg ha^−1^)	88	1.94	<0.0001	0.165	26	0.42	0.0290	0.024
Plant height (cm)	–	–	–	–	0.923	0.32	<0.0001	0.186
Ear height (cm)	0.513	0.27	<0.0001	0.044	0.403	0.23	<0.0001	0.044
Root lodging (%)	0.065	13.08	0.0089	0.025	−0.047	−2.65	0.0020	0.028
Stalk lodging (%)	0.225	2.20	<0.0001	0.072	0.032	0.42	0.2590	0.004
Ear rot (%)	0.011	0.14	0.5590	0.001	0.006	0.17	0.6390	0.001
Poor husk cover (%)	0.064	1.31	0.0002	0.036	0.039	0.92	0.0086	0.019
Turcicum leaf blight (1–5)	−0.026	−1.19	<0.0001	0.071	−0.004	−0.27	0.1280	0.008
Grey leaf spot (1–5)	0.024	1.01	<0.0001	0.087	−0.016	−0.81	0.0005	0.037

*Magnitude is the slope of the regression equation; ^α^percentage increases or decreases relative to the mean of the trait value.

The regression model accounted for variation ranging from 2.5% to 7.2% for agronomic traits and 3.6% to 8.7% for diseases in the PVT (**Table 5**). Similarly, the regression model accounted for 18.6%, 4.4%, 2.8%, and 3.7% for EH, PH, RL, and GLS, respectively, in the AVT. Results of genetic trends revealed an increase in PH of 0.92 cm year^−1^ at a rate of 0.32% year^−1^ in the PVT and AVT. On the other hand, an increase in EH of 0.51 cm year^−1^ (0.27% year^−1^) was observed for PVT genotypes, whereas AVT genotypes had an increase of 0.40 cm year^−1^ (0.23% year^−1^). Root lodging and SL increased at a rate of 13.8% year^−1^ and 2.2% year^−1^, respectively, in the PYT. In contrast, a rate of −2.65% year^−1^ was observed for RL in the AVT. There was a significant (p < 0.05) increase in HC at a rate of 0.064% year^−1^ and 0.92% year^−1^ in the PVT and AYT, respectively. Ear rot had a significant (p < 0.05) genetic gain of 0.14% year^−1^ with a mean of 7.8% and 0.011% year^−1^ with a mean of 3.3% for the PVT and AVT, respectively. Disease severity scores for TLB and GLS showed a significant (p < 0.05) decrease of −0.03 year^−1^ (−1.19% year^−1^) with a mean score of 2.3 and −0.02 year^−1^ (−0.81% year^−1^), and a mean score of 1.7, for PVT genotypes (TLB) and AVT genotypes (GLS), respectively. A distinction was recorded for GLS with PVT genotypes that had a significant (p < 0.05 increase in disease severity scores of 0.02 year^−1^ (1.01% year^−1^) and a mean score of 2.2.

### 3.3 Consistency in the performance of putatively superior maize genotypes

To assess the efficiency of the advancement process, the performance of some superior genotypes for GY that were advanced from the PVT to the AVT was tracked. The advancement of these entries from the PVT stage to the AVT stage was introduced in 2006 in the KHMP. These were the putatively superior genotypes (PSG), and their number ranged from one in 2019 to 20 in 2009 (**Table 6**). The average number of PSG advanced was 8.4 per year between 2006 and 2020. We compared the mean GY of the PSG in the PVT to the mean GY of all other PVT genotypes and to the commercial check hybrid H6213. Within the PVT, the PSG average GY is 114.7% of all other entries, ranging from 98% to 139.7%. The GY of the PSG in the PVT was, on average, 102% of the check hybrid H6213, with a range of 96.1 to 119%. A comparison of the GY of the PSG in the subsequent AVT to the mean GY of all other AVT entries and to the check H6213 was carried out. The PSG average GY in the AVT was 105.4% of all other genotypes, with a range of 97.7% to 118.8% (**Table 6**). The GY of the PSG in the AVT was, on average, 94.7% of the check H6213, with a range of 76.2% to 120.2%.

**Table 6:** A comparison of grain yield (kg ha^−1^) for putatively superior entries (PSG), other entries and the check variety (H6213) based on evaluations in the PVT and AVT between 2006 and 2019.

PVT year	(PSG) for advancement to AVT (*n*)	Grain yield (t/ha) in the PVT					AVT year	Grain yield (t/ha) in the AVT				
		Check-H6213	PSG	Other entries (OE)^1^	PSG vs. OE (%)	PSG vs. H6213 (%)		Check-H6213	PSG	OE^2^	PSG vs. OE (%)	PSG vs. H6213 (%)
2006	9		5,290	4,870	108.7		2007	5,770	5,490	5,190	105.7	95.1
2007	12	5,180	5,020	4,600	109.2	96.9	2008	7,120	5,420	5,500	98.6	76.2
2008	7	4,610	4,620	3,740	123.5	100.1	2009	5,980	5,870	5,540	105.9	98.2
2009	20	6,220	7,400	6,580	112.5	119	2010	8,240	6,990	6,560	106.7	84.9
2010	3	6,150	5,910	4,230	139.7	96.1	2011	7,570	6,690	5,630	118.8	88.4
2012	6	7,810	7,820	7,010	111.6	100.1	2013	4,910	5,900	6,040	97.7	120.1
2019	1	6,030	6,030	6,150	98	100	2020	7,010	7,010	6,690	104.7	100
Mean	8.29	6,000	6,010	5,310	114.7	102		6,660	6,200	5,880	105.4	94.7

PVT, Preliminary Variety Trials; AVT, Advanced Variety Trial. Other entries tested in the PVT and AVT are putatively superior genotypes (PSG), OE1, and OE2, respectively.

Firstly, the consistency of performance of the three PSG identified in the PYT of 2006 that were evaluated in the AYT of 2007 was assessed. These three genotypes had an average GY of 5,290 kg ha^−1^ in the PYT in 2006, whereas the mean of other entries in this stage was 4,870 kg ha^−1^. In the AYT of 2007, the three PSG had a consistently higher GY (5,490 kg ha^−1^) compared with the AYT mean of 5,190 kg ha^−1^ for the rest of the entries. Overall, the PSG yielded 1.15-fold more than other genotypes in the PYT, but the magnitude of difference (1.05-fold) relative to the other entries was marginally lower in the following year in the AYT.

Secondly, a comparison of the means of some specific genotypes meant for advancement in the PYT to the means of all other entries in the PYT trial was carried out. The advancement with the largest number of PSG (*n* = 20; 2009) in the PYT had a mean GY of 7,400 kg ha^−1^. These PSG were evaluated in the 2010 AYT and gave a mean GY of 6,990 kg ha^−1^, approximately 1.18-fold the yield of other entries in that trial but 12% less than the check (H6213). In 2012, the PSG had a mean GY of 7,810 kg ha^−1^ in PYT, and when these entries were evaluated in the 2013 AYT, they gave a mean GY of 5,900 kg ha^−1^, which was 2% less than the GY of other entries and 1.2-fold more than the commercial check hybrid. Based on the occurrence of the means of GY, the probabilities for having higher GY for PSG than the other entries each year were (GY_PSG_>GY_OE_) = 0.86 for PYT and *P*(GY_PSG_>GY_OE_) = 0.71 for AYT. However, the mean GY in the PYT PSG was not likely to be different from the commercial check hybrid, as *P*(GY_PSG_>GY_H6213_) = 0.5. Overall, the PSG were more likely to have a lower mean GY than the commercial check, as *P*(GY_PSG_>GY_H6213_) = 0.14) in the AYT (**Table 6**).

## 4 Discussion

It is a common practice in both the public and private sectors to use key performance indicators (KPIs), defined as quantifiable parameters, to ensure that activities are aligned toward achieving organizational goals through increasing transparency and accountability for overall long-term performance (Zakaria et al., 2011; Covarrubias-Pazaran, 2020). Thus, the number of varieties released by the public sector crop breeding programs has been frequently used as a KPI. However, varietal releases alone do not reflect the efficiency of a breeding program nor the impact of a breeding pipeline (Prasanna et al., 2022a). Estimates of genetic gains are an important KPI to measure the genetic progress, assess breeding efficiency, identify areas of improvement, and investment for accelerated genetic gains in delivering improved varieties to the farmers (Asea et al., 2023; Tarekegne et al., 2024).

The objective of this study was to estimate genetic gain for GY (kg ha^−1^), agronomic traits, and disease severity using historical data in the PVT (2003–2020) and AVT (1999–2020) variety trials conducted by the Kenya highland maize breeding program (KHMP), a program under Kenya Agricultural and Livestock Research Organization (KALRO). Previous genetic gain report for KHMP was published in the 1970s (Eberhart and Harrison, 1973).

The success of a crop breeding program mainly depends on the presence of genetic variation and the heritability of the traits under consideration. Broad-sense heritability was medium (>31%‒60%) for GY and HC in PVT and AVT. A similar heritability range was obtained for PH, EH, and SL in AVT. These results indicate that genetic progress for these traits can be achieved through careful selection and the use of simple selection methods like pedigree selection. In contrast, low heritability was recorded for RL, GLS, TLB, and ER in both AVT and PVT and PH and EH in the PVT, which suggests that improvement of these traits may be considerably difficult due to the masking effect of the environment on genotypic expression. This also shows the complex nature of inheritance of certain traits like resistance to some foliar diseases. Some of the traits with low heritability require selection methods that will progressively build desirable/favorable alleles to improve the population. The KHMP can improve the efficiency of selection for resistance to major diseases, especially ER, GLS, and TLB, by using artificial inoculation in trials at appropriate sites during hybrid evaluation.

In the current study, the general genetic gain estimates were higher in PVT relative to AVT across traits. The high genetic gain trend in PVT relative to AVT is mainly accounted for by the inbred lines in the PVT that are in early-stage generations (F_3_–F_5_), whereas the inbred lines in AVT are advanced (>F_6_). Genetic variance is usually higher in the early generations but reduces in the advanced generations due to selection. The GY genetic gain estimate for PVT was 88 kg ha^−1^ year^−1^, whereas that for AVT was 26 kg ha^−1^ year^−1^. While the germplasm is not similar, the genetic gain estimate in PVT in this study was comparable with the 81 kg ha^−1^ year^−1^ reported for Uganda (Asea et al., 2023), 62 kg ha^−1^ year^−1^ for Ethiopia Highland maize (Kebede et al., 2020), 61 kg ha^−1^ year^−1^ reported for the Zimbabwe National Breeding Program (Mazibuko et al., 2024), and 109.4 kg ha^−1^ year^−1^ CIMMYT hybrid breeding pipeline in Eastern and Southern Africa (Masuka et al., 2017). The GY genetic gain estimates of 26 kg ha^−1^ year^−1^ in the AVT was higher than that reported by the CIMMYT East Africa Product Profile-Highland breeding pipeline −70 kg ha^−1^ year^−1^ (Prasanna et al., 2022b). The lower genetic gain estimates in AVT relative to PVT may be due to the difference in the germplasm sample from the given testing stage. According to Covarrubia-Pazaran (2020), the use of early testing trials (within program management), i.e., PVT, and late testing trials (within program management), i.e., AVT to calculate genetic gain estimate uses different samples, but each will have different properties that affect the accuracy of the genetic gain estimate and Target Population Environment (TPE) coverage. The use of early-generation trials (PVT) provides a better estimate of the evolution of genetic variance, whereas advanced-stage genotypes provide a better estimate of the rate of genetic gain. In addition, the low annual genetic gain estimate in AVT genotypes may be attributed to the long crop growth cycle in the highland breeding program (Eberhart and Harrison, 1973). This calls for the adoption of DH technology to accelerate inbred line development.

Kenya Highland maize market segment has a defined Target Product Profile (TPP)^1^ (**Supplementary Table 1**), a blueprint for the design of new varieties that indicates the traits^2^ required in a new variety to meet or exceed the requirements of farmers, processors, and consumers (Donovan et al., 2022). As a result, agronomic traits in KHMP have been deliberately selected based on thresholds set for essential or nice-to-have traits for this TPP. The genotypes advanced from the PVT into AVT had a general trend of reduced ear height, thus indicating a deliberate selection strategy for the breeding program to advance genotypes with lower ear placement, an essential trait for Kenya’s highland maize market segment. A similar trend was also noted for RL and SL in PVT, which had increased genetic gain estimates relative to the decrease noted in AVT genotypes. This highlights that KHMP has made a concerted effort to select and advance genotypes from PVT to AVT with RL and SL threshold values lower than commercial check varieties, thus increasing the chances of releasing varieties that have good standability (root and stalk lodging). Furthermore, in collaboration with CIMMYT, KHMP has recently acquired elite inbred lines with short stature from the CIMMYT-Ethiopian Institute of Agricultural Research (EIAR) highland maize breeding program and has begun to introgress the trait into its breeding populations. This will eventually lead to greater use of parental inbred lines for a new generation of short-statured hybrids adapted to the Kenya highland agroecology.

The Kenya highland TPE (**Table 2**; **Figure 1**) is prone to ear and foliar diseases due to the conducive climatic conditions (high rainfall, high humidity, and temperature) for disease development. Consequently, KHMP has defined resistance to TLB, GLS, and ER as an essential trait, and this still needs continuous improvement. There was a trend toward increased resistance to TLB (−1.19% year^−1^) and GLS (−0.81% year^−1^) in the AVT, indicating that the defined selection strategy for these diseases and advancement decision from PVT to AVT was optimal. In the PVT, genotypes had reduced GLS resistance, as evidenced by increased severity scores of 0.02 year^−1^ (1.01% year^−1^). The inconsistency may be due to the use of natural disease sites as opposed to artificial inoculation. Lack of adequate pathogen pressure may lead to reduced disease incidence in some locations and, hence, low disease severity scores (Kenworthy, 1966; Ward et al., 1999; Jakhar et al., 2017; Njeru et al., 2023). Without artificial inoculation, screening maize at disease hotspots would be the best alternative to attain high disease pressure, thus enhancing genetic gain during selection (Lopez-Zuniga et al., 2018).

Good husk cover (tip of the ear fully covered to ensure restricted entry and damage by water, opportunistic insects, birds, and pathogens) is an essential trait in the KHMP breeding pipeline (**Supplementary Table 1**). The results indicated little progress in the reduction of the incidence of poor husk cover in both PVT 1.31% year^−1^ and AVT (0.92% year^−1^). KHMP should consider infusing germplasm with good husk coverage from other programs, especially CIMMYT, to enhance gains for husk cover in the desired direction. Development of varieties with a good husk cover would be valuable because the ongoing climate change will lead to an increase in temperature, and hence, maize will be cultivated in warmer conditions, which favors maize ear rot infections (Warfield and Davis, 1996; Miedaner and Juroszek, 2021; Nnadi and Carter, 2021). Poor husk cover is related to the high incidence of ear/cob rots and insect pests like Fall Armyworms (Prasanna et al., 2022a). Enhancing resistance to ER would improve grain quality and prevent contamination by mycotoxins, which are harmful to livestock and humans and are widespread in Kenya (Ajanga and Hillocks, 2000; Mutiga et al., 2017).

The analysis of the GY of PSG which were advanced to the AVT showed that while they had a GY of 114.7% of all other PVT genotypes, they had a GY of just 105.4% of other AVT, and they were just 94.7% of the GY of the commercial check hybrid H6213 in the AVT. There is a significant genotype × year interaction affecting advancements to the AVT, yet this seems to have had less effect on advancements to the PVT. The average GY of the PVT experiments was 26% lower than the average GY of the AVT. It is possible that performance in low-GY sites may not translate to GY in high-GY sites. Adopting accurate and efficient phenotyping strategies, with optimal replication over years in environments with known levels of reproducibility for target agroecological zones, could enhance the efficiency in selection for advancement (Duvick, 2005; Gunundu et al., 2023). The focus of the KHMP breeding pipeline has been to improve GY, which averaged 4,530 kg ha^−1^ in the PVT and 6,100 kg ha^−1^ in the AVT. The mean differences in GY between the two evaluation stages suggest the superiority of the fraction of genotypes selected for advancement in terms of GY. Although there were significant, positive genetic gain estimates for GY in the PVT and AVT, most of the putatively superior genotypes (or advanced hybrids) yielded less than the commercial check hybrid (H6213) released by the Kenya Seed Company in 2002.

In this study, most of the genotypes selected in the PVT and advanced in the breeding pipeline evaluation stages had lower GY than the commercial check hybrid. While the primary aim of this study was to document current genetic trends within the KALRO highland maize breeding pipeline, the compilation of historical datasets provides the opportunity to identify key areas to improve breeding efficiency. The introduction of germplasm from similar agroecological zones in Central and South America played a major role in the establishment of the KHMP breeding pipeline for the Kenyan highlands agroecology (Harrison, 1970). Similar to the CIMMYT-Ethiopia highland maize breeding program, the genetic pool in the KALRO highland maize program still relies on parental inbred lines developed from Ecuador-573, Kitale-SYN, and Pool-9A (Prasanna et al., 2022a). Genetic trends in hybrid maize breeding pipelines are a function of the gains in female and male parental inbred lines. The pedigree data for the current study showed that most of the male inbred lines in the single-cross female parents of the three-way hybrids remained the same throughout the study, with only six male parents of the single-cross hybrid female parents accounting for 75% of all candidate hybrids tested in the AVT. Five of these males of single-crosses were present in hybrids in the first year of testing and were still key male parental inbred lines in hybrids in 2020, and only one male of the single-crosses first entered the AVT in 2006. The observed low performance of the PSG suggests that for the program to move beyond the current GY performance ceiling, it must utilize international networks to revamp the breeding pipeline with superior exotic elite inbred lines, which are adapted to similar agroecologies (Harrison, 1970; Atlin et al., 2017), beyond the original introductions from Central and South America that were used to initiate hybrid maize development in the Kenyan highlands (Harrison, 1970). The findings of this two-decade study will enable the program to reorient its breeding strategy strategically by adopting more efficient modern breeding operations for better resource utilization and enhanced genetic gains.

To further enhance the genetic trends across all important traits in the TPP, several practical breeding modernization approaches could be adopted in the KHMP, as outlined below:

- Adopt a product profile-based breeding approach, where the efforts are aimed at developing products for the current market demands. Market-led breeding operations could compel the program to adopt cost-effective methods that would fast-track the release of improved varieties. To identify the cost drivers for the breeding operations and the potential ways of optimizing breeding metrics, the program has participated in the costing of its pipeline using the University of Queensland Breeding Costing Tool (UQBCT) through the CGIAR Accelerated Breeding Initiative (ABI).
- Implementation of doubled haploid (DH) technology for faster development of genetically homozygous inbred lines and accelerated hybrid development (Chaikam et al., 2019). CIMMYT uses DH technology for line development, and KHMP can use the service, with the cost covered through special projects.
- Using an artificial disease screening facility for TLB, GLS, and MLN will ensure accurate selection and advancement of genotypes with these essential traits for KHMP. KALRO utilizes the CIMMYT-operated MLN Screening Facility at Naivasha and an artificial foliar disease screening facility at Kakamega in Kenya. These facilities could potentially be used for KHMP.
- Adoption of sparse testing for breeding trials: Field phenotyping is one of the major cost drivers in most of the breeding programs (Lane et al., 2020). Sparse testing can enhance the evaluation of many entries across multiple environments, improving breeding efficiency, and optimizing use of resources. In sparse testing for multienvironment breeding trials, not all genotypes of interest are grown in each environment but the alleles of interest are tested across specific environments based on prior genotyping data (Atanda et al., 2022). Sparse testing could be coupled with an expansion in the testing network within East Africa while leveraging the leadership of international collaborative partners like CIMMYT in Africa. Increasing the size of the testing network is a key driver of enhancing genetic gains in maize yields in the US (Cooper et al., 2014) and Eastern and Southern Africa (Masuka et al., 2017; Prasanna et al., 2022b).
- Digitization of breeding program operations, i.e., electronic data capturing and use of data management systems like Breeding Management System (BMS) or the Enterprise Breeding System (EBS), will enhance data turnaround time and data quality for data-driven selection decisions and advancement of genotypes within the breeding pipeline.
- Adoption of molecular markers for forward breeding to integrate qualitative traits and genomic selection for simultaneous improvement of multiple traits (Moose and Mumm, 2008). Forward breeding can be used for selection to enrich populations for favorable alleles before field phenotyping. Coupling forward breeding with genomic selection to simultaneously improve the germplasm for multiple traits, including those that are inherited quantitatively, such as GY, root, and stalk lodging, ear rot, GLS, TLB, and MLN would be an excellent strategy to increase genetic gains (Poland et al., 2009; Larkin et al., 2019). KHMP can leverage current efforts by CIMMYT’s Global Maize Program to implement molecular breeding in conjunction (Crossa et al., 2014; Prasanna et al., 2022b; Omondi et al., 2023).

## 5 Conclusions

The Kenya Highland Maize Program (KHMP) focuses on breeding and deploying elite, climate-resilient improved maize hybrid varieties. This study estimated genetic gains for grain yield (GY), agronomic traits, and disease resistance traits in the PVT and AVT conducted between 1999 and 2020. The study revealed significant genetic gains for GY, EH, PH, HC, and disease resistance (GLS and TLB). Thus, the KHMP has made desired strides in hybrid maize development over the years, resulting in developing and releasing highland maize varieties that are productive, short stature, and disease-resistant. The genetic gain trends for some traits were not consistently in the desired direction. To further improve the genetic gains, the KHMP plans to adopt a product-profile-based breeding approach, breeding methods, and technologies that can increase breeding efficiency and genetic gains.

## Data availability statement

The datasets presented in this study can be found in online repositories. The names of the repository/repositories and accession number(s) can be found in the article/**Supplementary Material**.

## Author contributions

DL: Data curation, Investigation, Methodology, Writing – original draft, Writing – review & editing. ES: Conceptualization, Formal analysis, Methodology, Software, Writing – original draft, Writing – review & editing. BA: Conceptualization, Data curation, Visualization, Writing – original draft. CS: Methodology, Project administration, Resources, Visualization, Writing – original draft, Writing – review & editing. WC: Formal analysis, Investigation, Resources, Validation, Writing – review & editing. LM: Conceptualization, Data curation, Resources, Supervision, Visualization, Writing – original draft, Writing – review & editing. DMa: Conceptualization, Formal analysis, Funding acquisition, Methodology, Validation, Writing – review & editing. MM: Data curation, Formal analysis, Validation, Writing – review & editing. DMi: Formal analysis, Methodology, Resources, Writing – review & editing. SM: Formal analysis, Investigation, Visualization, Writing – review & editing. AL: Data curation, Formal analysis, Visualization, Writing – review & editing. BD: Funding acquisition, Investigation, Supervision, Validation, Writing – review & editing. BP: Funding acquisition, Investigation, Project administration, Supervision, Writing – review & editing.

## Footnotes

1

*https://glomip.cgiar.org/target-product-profiles*

2

*https://www.cimmyt.org/blogs/market-segmentation-and-target-product-profiles-tpps-developing-and-delivering-impactful-products-for-farming-communities/*
