# Supplementary material for: Genetic trends in the Kenya Highland Maize Breeding Program between 1999 and 2020
Source: Front Plant Sci. 2024 Jul 1;15:1416538. doi: 10.3389/fpls.2024.1416538 (PMC11246847; doi:10.3389/fpls.2024.1416538)
Supplement: Supplementary file 1 [file Table_1.docx]

Supplementary Material

| ***Target Product Profile*** | | | | | |
| --- | --- | --- | --- | --- | --- |
| ***Late-maturing white maize hybrids adapted to the Eastern African rainfed highlands are suitable for food use.*** | | | | | |
| ***Intermediate/ Late maturing, Food, and feed (Dual purpose), Grain colour white, Rainfed, Flint/Semi-dent grain texture*** | | | | | |
| ***Maize*** | | | | | |
| ***East Africa*** | | | | | |
| ***East Africa*** | | | | | |
| ***Kenya*** | | | | | |
| ***770,000 hectares*** | | | | | |
| ***Hybrids*** | | | | | |
| ***Highland/Kitale, Altitude >1800masl, Rainfall >1200 mm, Highland (FAO 700 series)*** | | | | | |
| ***Kenya Highlands: March-December (main season), October to March (Short/off-season)*** | | | | | |
| ***Trait*** | ***Scale*** | ***Minimum Score*** | ***Trait requirement*** | ***Improve trait*** | ***Threshold trait*** |
| *Flour recovery* | *%* | *85* | *Essential* |  | *Y* |
| *Chalatinization* | *visual* | *Subjective score* | *Nice-to-have* |  | *Y* |
| *Green mealies* | *taste* | *Sweet taste (subjective taste)* | *Nice-to-have* | *Y* |  |
| *Yield under optimum conditions* | *tons/ha* | *At least 5% greater than the relevant benchmark commercial check* | *Essential* | *Y* |  |
| *Yield under drought stress* | *tons/ha* | *At least 5% greater than the best trait check (internal/ external)* | *Value added* | *Y* |  |
| *Yield under low N stress (nitrogen use efficiency or NUE)* | *tons/ha* | *At least 5% greater than the best trait check (internal/ external)* | *Value added* | *Y* |  |
| *Lodging resistance (stalk)* | *%* | *Under optimal - <10% lodging or at par with (mean) commercial checks* | *Essential* | *Y* |  |
| *Lodging resistance (root)* | *%* | *Under optimal - <10% lodging or at par with (mean) commercial checks* | *Essential* | *Y* |  |
| *Moisture at harvest* | *%* | *Equivalent to best check or <20% at harvest* | *Essential* | *Y* |  |
| *ASI* | *days* | *<5 days under drought* | *Essential* |  | *Y* |
| *Stay-green* | *1 to 10* | *≤ 6.0 Senescence score at physiological maturity* | *Nice-to-have* |  |  |
| *Plant height* | *cm* | *<10 cm of the best check* | *Essential* | *Y* |  |
| *Ear position* | *ratio* | *<0.5* | *Essential* | *Y* |  |
| *Ear aspect* | *1 to 5* | *≤3.0 Ear Aspect score or at par with (mean) commercial checks* | *Essential* | *Y* |  |
| *Tip-filling* | *1 to 5* | *≤3.0 Ear Aspect score or at par with (mean) commercial checks* | *Nice-to-have* |  | *Y* |
| *Soil acidity* | *1 to 9* | *<4 Soil acidity score* | *Nice-to-have* |  | *Y* |
| *Gray Leaf Spot (GLS) resistance* | *1 to 9* | *≤4.0 GLS score* | *Essential* | *Y* |  |
| *Fusarium Ear Rot (FER) resistance* | *%* | *Less than 10% incidence* | *Essential* | *Y* |  |
| *Maize Lethal Necrosis (MLN) resistance* | *1 to 9* | *MLN score of 4.0 or less* | *Nice-to-have* | *Y* |  |
| *Yield under artificial MLN inoculation* | *tons/ha* | *at least 4 t/ha under artificial MLN inoculation* | *Nice-to-have* | *Y* |  |
| *Maize Streak Virus (MSV) resistance* | *1 to 9* | *≤4.0 MSV score* | *Essential* | *Y* |  |
| *Common Rust (PS) resistance* | *1 to 9* | *≤4.0 PS score* | *Essential* | *Y* |  |
| *Turcicum Leaf Blight (TLB) resistance* | *1 to 9* | *≤4.0 TLB score* | *Essential* | *Y* |  |
| *Production split/synchronization* | *Days* | *±3* | *Essential* |  | *Y* |
| *Inbred line grain yield* | *t/ha* | *>2* | *Essential* |  | *Y* |
| *Single-cross parent grain yield* | *t/ha* | *>4* | *Essential* |  | *Y* |
| *Male plant height: female ear height ratio* |  | *>1* | *Essential* |  | *Y* |
| *Commercial checks: H614D, H6213, KH600 23A, DK777, H6218* | | | | | |

# Supplementary Table 1. KALRO Highland maize breeding program market segment and Target Product Profile (TPP)
